# The cell-type-specific roles of Toll-like receptors in herpes simplex virus infection and pathogenesis

**DOI:** 10.1080/21505594.2026.2664934

**Published:** 2026-04-24

**Authors:** Haifeng Yu, Lina Wang, Jun Gong, Helin Lu, Wu Liu, Liqiong Ding

**Affiliations:** a School of Pharmacy, Hubei University of Science and Technology, Xianning, China; b Office of Drug Clinical Trial Institution, Xianning Central Hospital, The First Affiliated Hospital of Hubei University of Science and Technology, Xianning, China

**Keywords:** Toll-like receptors, HSV, NF-κB, inflammatory factor, interferon

## Abstract

Herpes simplex virus (HSV) initiates infection in mucosal epithelial cells, forming characteristic lesions before establishing lifelong latency in sensory neurons. The innate immune response to HSV is critically mediated by the Toll-like receptor (TLR) family. Expressed in a cell‑type‑specific manner, TLRs recognize viral components, leading to the production of interferons and pro-inflammatory cytokines. Different cell populations, by virtue of their unique TLR expression profiles and signaling contexts, mount specialized and non-redundant responses to the same viral pathogen. We systematically synthesize current research to elucidate the roles of specific TLRs in major cellular targets of HSV, including mucosal epithelial cells, fibroblasts, plasmacytoid dendritic cells, macrophages, peripheral neurons, and resident central nervous system cells. By examining how TLR-mediated sensing and signaling diverge across this cellular landscape, this article provides an integrated framework for understanding the coordinated, multi-layered immune defense against HSV and highlights the implications for pathogenesis and therapeutic strategies.

## Introduction

Herpes simplex virus (HSV), a highly prevalent double-stranded DNA virus, encompasses two primary types: HSV-1 and HSV-2. HSV-1 infects approximately 80% of the global population, establishing a lifelong infection that can manifest from asymptomatic carriage to recurrent mucocutaneous lesions and, in rare but severe cases, encephalitis [1,2]. After infecting epithelial cells and fibroblasts, HSV-1 enters peripheral sensory neurons and establishes latency primarily within the trigeminal ganglia. Periodic reactivation can lead to recurrent lesions or, upon spread to the central nervous system (CNS), life-threatening herpes simplex encephalitis (HSE) [3,4]. HSV-2, though less common globally, is a major cause of genital ulcer disease and similarly establishes latency in sacral ganglia. Notably, HSV-2 infection significantly enhances the risk of HIV-1 acquisition and transmission, adding to its global health burden [5,6].

The host innate immune system employs a sophisticated array of pattern recognition receptors to detect invading pathogens [7]. Among these, Toll-like receptors (TLRs) play a pivotal role as frontline sensors for microbial components. TLRs are broadly categorized by their cellular localization and ligand specificity: cell-surface TLRs such as TLR2 and TLR4 recognize viral structural proteins, while endosomal TLRs, including TLR3, TLR7, and TLR9, are specialized for detecting viral nucleic acids-double-stranded RNA (dsRNA), single-stranded RNA (ssRNA), and unmethylated CpG DNA, respectively [8–10]. Upon ligand engagement, TLRs initiate signal transduction primarily through two adaptor-dependent pathways: the Myeloid differentiation primary response 88 (MyD88)-dependent pathway and the TIR-domain-containing adapter-inducing interferon-β (TRIF)-dependent pathway. MyD88 is the universal adaptor utilized by all TLRs except TLR3 [11]. Its recruitment initiates a signaling cascade that culminates in the activation of nuclear factor-κB (NF-κB) and, in certain cellular contexts, interferon regulatory factor 7 (IRF7). In contrast, the TRIF pathway is employed by TLR3 and, as an additional pathway, by TLR4 [12]. TRIF activation leads to the phosphorylation and nuclear translocation of interferon regulatory factor 3 (IRF3), which predominantly induces the expression of interferon-β (IFN-β) and a distinct set of interferon-stimulated genes (ISGs).

The MyD88- and TRIF-dependent pathways collectively drive the expression of pro-inflammatory cytokines and type I interferons (type I IFNs), which are crucial for establishing an antiviral state and orchestrating subsequent adaptive immunity [13–15]. Notably, the utilization of these pathways is highly cell-type-specific, depending on the expression profile of TLRs and the functional specialization of each cell. The TLR3-TRIF axis, responsible for sensing dsRNA produced during viral replication, exhibits significant cell-intrinsic and species-specific variations in its antiviral activity [16]. The critical importance of TLR3 in CNS defense is underscored by the discovery that genetic defects in the TLR3 pathway are strongly associated with increased susceptibility to childhood HSE [17–19]. Also, a clinical report documented that a young adult carrying a TLR4 mutation developed bilateral sequential HSV-2 panophthalmitis, resulting in severe vision loss, suggesting that TLR4 May be associated with HSV ocular inflammation [20].

**Figure 1.**
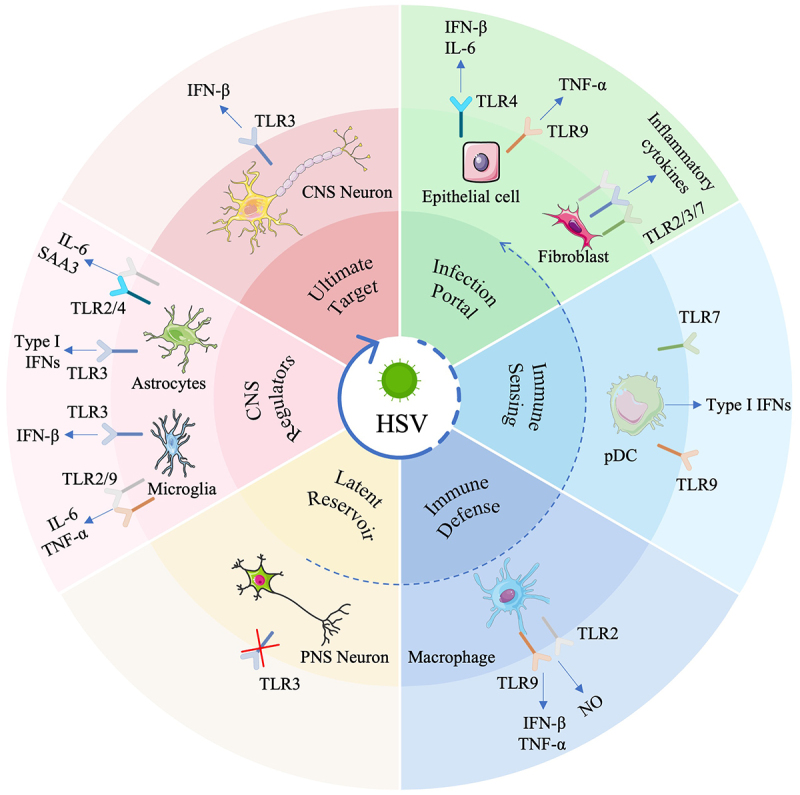
Schematic diagram of the role of tlr in HSV infection across different cell types. Epithelial cells, as the initial viral entry portals, utilize TLR4 and TLR9 to initiate local antiviral responses. Fibroblasts also contribute to early antiviral defense through TLR2, TLR3, and TLR7. pDcs rely on endosomal TLR7 and TLR9 to become primary producers of type I IFNs. Macrophages employ TLR9 and TLR2 to mediate viral recognition and exert antiviral effects. Trigeminal ganglion neurons exhibit attenuated TLR3-dependent immune responses, a feature that may contribute to the establishment of viral latency. In the central nervous system, microglia engage TLR3 for antiviral defense and TLR2/9 for pro-inflammatory responses, whereas astrocytes utilize TLR3 for antiviral defense and TLR2/4 to drive neuroinflammation. TLR3 in cortical neurons provides intrinsic antiviral defense and is a key determinant of susceptibility to HSE [23,24].

A central and emerging paradigm in HSV immunology is that the immune outcome of infection is not dictated by a single receptor or cell type but is instead determined by a complex, spatially organized network composed of diverse cell populations, each equipped with a distinct TLR expression profile and functional specialization [21,22]. Mucosal epithelial cells and fibroblasts serve as the primary sites of viral entry, initiating local antiviral responses [23]. Specialized immune cells, including plasmacytoid dendritic cells (pDCs) and macrophages, act as key mediators of systemic immunity [24], whereas within the central nervous system (CNS), microglia, astrocytes, and neurons engage distinct TLR repertoires to determine the outcome of infection, ranging from viral clearance to latency and encephalitis (Figure 1).

Therefore, this review aims to synthesize current knowledge by proposing and elaborating on a central thesis: the immunological resolution of HSV infection involves a coordinated network of diverse cell types, each playing a distinct role through their cell-specific TLR expression patterns and signaling capabilities. We will systematically examine the roles of TLRs in major cell targets of HSV, including epithelial cells, fibroblasts, dendritic cells, macrophages, peripheral neurons, and CNS-resident cells (microglia, astrocytes, and central neurons), highlighting how their discrete TLR-driven responses are integrated to shape the overall host defense, from initial containment and establishment of latency to pathological inflammation. By integrating epidemiological, clinical, and mechanistic insights, this analysis provides a holistic framework for understanding HSV pathogenesis and informs the rational design of TLR-targeted therapeutic and preventive strategies.

## The role of TLRs in different cells in HSV infection

The immune response to HSV infection is mediated by a diverse array of cell types, each employing a distinct repertoire of TLRs to recognize the virus and initiate appropriate downstream responses. The following subsections provide a detailed discussion of TLR expression and function in each relevant cell type.

### Infection portal and initial replication-mucosal epithelial cells and fibroblasts

In epithelial cells and fibroblasts, TLRs serve as key molecules for recognizing HSV and initiating early innate immune responses [25]. Different TLR members cooperate through specific signaling pathways to establish an antiviral state.

**Figure 2.**
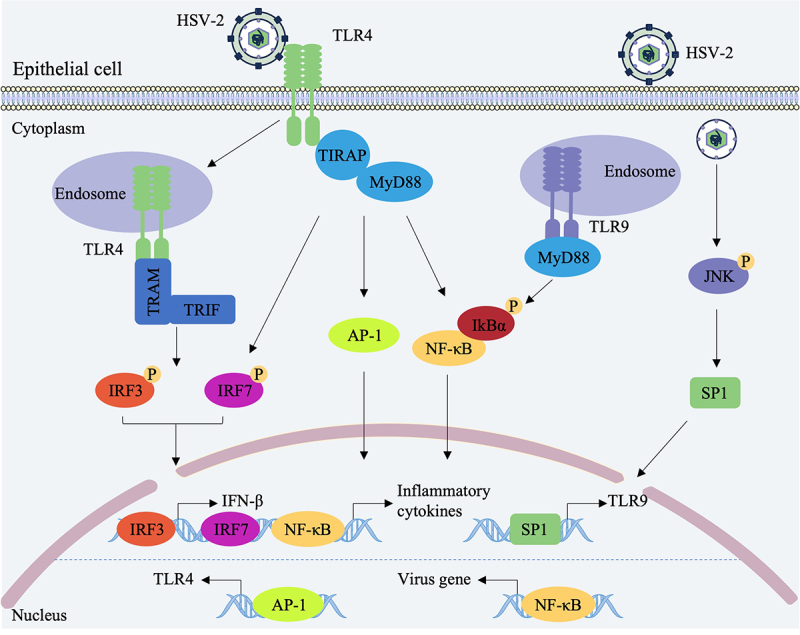
The involvement of TLR4 and TLR9 in epithelial cells in HSV infection. HSV-2 infection upregulates the expression of TLR4 in epithelial cells and activates NF-κB and IRF3/IRF7, promoting the production of inflammatory cytokines and type I IFNs [26,27]. HSV-2 can also upregulate the expression of TLR9 in epithelial cells, effectively stimulating innate immunity to control the infection [29]. The upregulation of TLR9 is related to the SP1/JNK signaling pathway. In certain contexts, sustained TLR4 activation triggers a positive feedback loop via AP-1, which binds to the TLR4 promoter and upregulates TLR4 expression, thereby contributing to viral replication [28]. Likewise, TLR9-mediated NF-κB activation can be exploited by HSV-2 to enhance viral gene transcription [31].

Multiple TLRs participate in the recognition and response to HSV in epithelial cells. HSV-2 infection upregulates TLR4 expression in cervical epithelial cells and activates NF‑κB along with the phosphorylation of IRF3 and IRF7, thereby promoting the production of IL-6 and IFN-β in a TLR4‑dependent manner [26,27] (Figure 2). In addition to its role in antiviral defense, TLR4 signaling is also exploited by HSV-2 to facilitate viral replication. In genital epithelial cells, HSV-2 infection triggers a positive feedback loop via AP-1, a transcription factor activated downstream of TLR4. AP-1 binds to the TLR4 promoter, upregulating TLR4 expression and leading to sustained TLR4 signaling [28]. Knockdown of TLR4 significantly impairs HSV-2 replication, indicating that TLR4 signaling is required for efficient viral replication. This seemingly paradoxical role of TLR4 might be explained by temporal dynamics. Early after infection (4–6 h), TLR4 activation induces IFN-β and pro-inflammatory cytokines through the TRIF-IRF3 and MyD88-NF-κB pathways, contributing to an initial antiviral state [26,27]. However, as infection progresses (24 h), sustained TLR4 activation triggers a positive feedback loop via AP-1, which upregulates TLR4 expression and promotes viral replication [28].

TLR9 also contributes to epithelial anti‑HSV immunity. HSV-2 infection upregulates the expression of TLR9, MyD88, and NF‑κB in HaCaT cells, effectively stimulating innate immunity to control infection [29] (Figure 2). In genital epithelial cells, HSV-2 promotes TLR9 transcription via the SP1/JNK signaling pathway. Upon infection, transcription factor SP1 translocates to the nucleus and binds to the TLR9 promoter, whereas inhibition of the JNK pathway blocks SP1 nuclear translocation and reduces TLR9 expression [30]. The relationship between TLR9 signaling and HSV replication, however, appears bidirectional. A study using the TLR9 inhibitor dihydromyricetin demonstrated that suppressing TLR9 expression in Vero cells reduces HSV‑1 replication, indicating that TLR9 signaling is required for efficient viral replication in that context [31]. This apparent discrepancy may be attributed to differences in viral serotypes (HSV-2 vs. HSV-1) and cell models (human genital epithelial cells vs. Vero cells). In addition to these experimental variables, the dual role of TLR9 reflects viral exploitation of host signaling pathways. NF-κB binding sites have been identified in the promoters of several HSV genes, and NF-κB activation directly enhances viral gene transcription [32,33]. Thus, while TLR9-mediated NF-κB activation contributes to host inflammatory responses, the virus can simultaneously utilize this pathway to facilitate its own gene expression. In this context, HSV-induced upregulation of TLR9 May represent a viral mechanism to create a replication-favorable environment, with the accompanying inflammatory response representing a host counter-measure that, under certain experimental conditions, results in reduced viral load [29]. Whether TLR9 activation suppresses or supports viral replication is therefore determined by the balance between host-mediated antiviral effects and viral exploitation of the same pathway.

TLR signaling is likewise broadly activated in fibroblasts [34]. Transcriptomic analysis indicates that HSV-1 infection of human gingival fibroblasts significantly upregulates TLR2, TLR3, TLR7 and their downstream IFNAR‑JAK‑STAT signaling pathways [35], suggesting that TLR‑mediated recognition plays an important role in the innate immune response of fibroblasts against HSV.

In parallel with the host’s attempts to amplify TLR expression for antiviral defense, HSV has evolved countermeasures to evade this detection at the epithelial surface. A notable example is the virion host shutoff (vhs) protein of HSV-2. In human vaginal epithelial cells, infection with wild-type HSV-2, but not a vhs-null mutant, significantly downregulates the expression of TLR2 and TLR3 at both the mRNA and protein levels [36]. This suppression extends to the cytosolic sensors RIG-I and MDA-5, effectively reducing the cell’s ability to sense viral components. Consequently, vhs inhibits the activation and nuclear translocation of IRF3 and dampens the subsequent production of IFN-β, thereby facilitating viral replication at the initial site of infection.

In summary, TLR4 and TLR9 establish a multi‑layered sensing and defense network against HSV in epithelial cells, providing a critical foundation for suppressing early viral replication. However, HSV has evolved countermeasures to subvert this host defense. The virus exploits TLR4-mediated AP-1 activation and TLR9-mediated NF-κB activation to create a replication-favorable environment [28,31]. In addition, HSV actively suppresses immune detection through the vhs protein, which downregulates TLR2 and TLR3 expression [36]. This dynamic interplay between host TLR sensing and viral immune evasion determines the outcome of HSV infection at the epithelial barrier.

### Early immune sensing by plasmacytoid dendritic cells

Plasmacytoid dendritic cells are specialized immune cells that serve as primary producers of type I IFNs during HSV infection. These cells detect HSV through endosomal TLR7 and TLR9, which recognize ssRNA and unmethylated CpG DNA motifs present in the HSV genome, respectively [37–39].

Upon ligand engagement, TLR7 and TLR9 recruit the adaptor protein MyD88 to form a signaling complex containing TRAF6 and IRF7, leading to direct phosphorylation and activation of IRF7 [40]. IRF7 serves as the central transcription factor driving the expression of multiple IFN-α subtypes. The MAP3K kinase MEKK3 has been identified as an essential activator of IRF7 within this pathway; *in vivo* knockdown of MEKK3 impairs type I IFNs induction and increases susceptibility to HSV‑1 infection in mice [41].

The TLR9-dependent mechanism of pDCs is particularly important in localized HSV infections. During HSV-1 keratitis, resident pDCs constitutively present in the normal cornea increase in number and become the main source of IFN‑α in the corneal stroma [42]. Through TLR9 signaling, these pDCs produce abundant type I IFNs and also prevent the reprogramming of regulatory T cells into pre‑effector Tregs. Local depletion of pDCs leads to severe clinical disease, underscoring the central role of pDC-mediated, TLR9-dependent HSV sensing in local antiviral defense.

In summary, pDCs primarily recognize HSV through TLR7 and TLR9 and initiate type I IFNs responses via the MyD88‑IRF7 axis, with TLR9 playing a particularly important role in local infections such as HSV keratitis. These cells are indispensable for controlling early viral spread and modulating the local immune microenvironment.

### Innate immune defense and clearance-macrophages

Macrophages serve as key innate immune effectors in the anti-HSV response. Single‑cell analyses of HSV encephalitis models reveal that monocyte/macrophage‑like cells are the predominant infected cell type, infiltrating the brain beginning on day 4 post‑infection and peaking on day 7 [43]. Depleting macrophages impairs early viral control and increases mortality in mice [44], underscoring the critical role of these cells in host defense against HSV.

In murine macrophages, TLR9 plays a central role in viral recognition. TLR9 activation induces the expression of IFN-β and TNF-α genes through a MyD88 adapter-like (Mal)-dependent mechanism, with ERK1/2 kinases identified as key regulators [45]. MyD88^–/–^Trif^–/–^ double‑deficient cells show significantly reduced ISGs induction, indicating that these adaptors contribute to TLR-mediated antiviral gene programs in macrophages [46]. It is worth noting that while TLR9-mediated responses are protective in many contexts, excessive or sustained activation of TLR signaling in macrophages can contribute to immunopathology. Dysregulated macrophage activation during HSV encephalitis can exacerbate neuroinflammation and tissue damage [44], highlighting that the outcome of TLR activation is determined by the intensity and duration of the inflammatory response.

In addition to TLR9, evidence from murine models suggests that TLR2 also contributes to macrophage-mediated antiviral functions. Studies using TLR2/9-deficient mice demonstrate that these receptors collectively mediate macrophage responses in the trigeminal ganglia, including iNOS-mediated nitric oxide production, which exerts direct antiviral effects [47,48].

Beyond the host’s utilization of TLR signaling for antiviral defense, HSV has evolved strategies to actively subvert these pathways in macrophages and related myeloid cells. The immediate-early protein ICP0 plays a pivotal role in this immune evasion. Functional studies using murine peritoneal macrophages have demonstrated that infection with ICP0-null mutant viruses results in significantly elevated IL-6 production compared to wild-type HSV-1, an effect that is strictly dependent on TLR2 [49]. Mechanistically, ICP0 antagonizes TLR2 signaling by targeting the essential downstream adaptor proteins MyD88 and Mal for proteasomal degradation. This activity requires the RING finger domain of ICP0 and its associated E3 ubiquitin ligase function, which together mediate the specific elimination of these adaptors, thereby blocking NF-κB activation and the subsequent inflammatory cytokine cascade. In addition to ICP0-mediated inhibition of TLR2 signaling, studies in human monocytic cells have revealed that HSV-1 targets TLR3 responses within the myeloid lineage. Using the U937 monocytic cell line, Peri et al. demonstrated that the HSV-1 Us3 protein kinase is involved in modulating TLR3-mediated antiviral immunity. Infection with a Us3-deletion virus resulted in significantly increased TLR3 mRNA and protein expression, enhanced IRF3 dimerization, and elevated type I IFNs production compared to wild-type virus [50]. These findings suggest that Us3 functions to suppress TLR3-dependent signaling in cells of the monocyte/macrophage lineage, complementing the ICP0-mediated inhibition of TLR2 signaling.

In summary, macrophages are critical effector cells in anti-HSV immunity, serving as the primary target of infection and executing TLR-dependent antiviral programs. TLR9 serves as the primary sensor for HSV DNA in murine macrophages, while TLR2 contributes alongside TLR9 to antiviral functions. However, HSV-1 has evolved potent countermeasures to subvert these TLR-mediated defenses. ICP0 disarms TLR2 signaling by promoting the proteasomal degradation of MyD88 and Mal [49], while Us3 suppresses TLR3 responses in monocytic cells [50]. Together, these viral strategies enable HSV-1 to evade macrophage-mediated immunity, highlighting the central role of macrophages in the host-virus interaction.

### Viral dissemination and latent reservoir-peripheral sensory neurons

The role of TLRs in HSV infection of neurons is complex and cell-type-specific. Trigeminal ganglion neurons serve as a classic site for HSV-1 latency. These neurons exhibit attenuated TLR3-dependent immune responses, a feature that may contribute to the establishment and maintenance of viral latency by reducing the likelihood of immune-mediated viral clearance [51]. This likely represents one of several factors that collectively govern the latent state [52].

**Figure 3.**
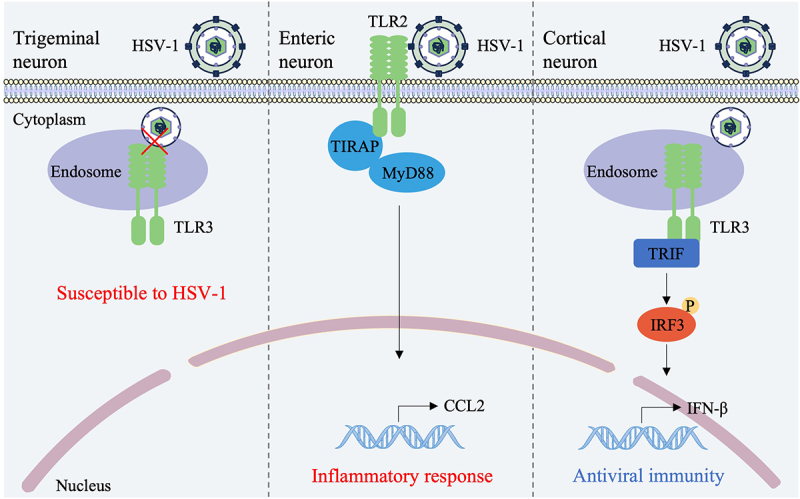
The involvement of TLR2 and TLR3 in HSV-infected neurons. Trigeminal ganglion neurons exhibit limited TLR3-dependent intrinsic immune responses against HSV in contrast to cortical neurons [51]. HSV-1 infection triggers TLR2-MyD88 signaling in enteric neurons, mediating the production of CCL2 and inducing structural and functional changes in the enteric nervous system [53,54]. The TLR3 signaling pathway maintains the constitutive expression level of IFN-β in cortical neurons, conferring antiviral properties [63].

Beyond its role in latency, TLR signaling in peripheral neurons contributes to local pathology. In enteric neurons, HSV-1 infection triggers TLR2-MyD88 signaling, which mediates the production of chemokines such as CCL2, coordinates macrophage recruitment, and induces structural and functional alterations within the enteric nervous system [53,54] (Figure 3). Studies in TLR2 knockout mice show milder HSV-1-induced gastrointestinal motility disorders, indicating that TLR2-mediated responses can exacerbate pathogen-induced neuromuscular dysfunction. This observation highlights the dual nature of TLR activation in peripheral neurons: while TLR signaling contributes to antiviral defense in some contexts, excessive or dysregulated activation can drive immunopathology.

### Microglia and astrocytes: innate immune cells of the central nervous system

Upon crossing the blood-brain barrier and entering the CNS, HSV encounters the brain’s resident innate immune cells, microglia and astrocytes. These cells form the first line of immune defense through pattern recognition receptors, including TLRs, which play key roles in the response to HSV infection.

Microglia, the resident macrophages of the CNS, express a broad TLR repertoire and serve as central sensors for HSV recognition. Studies have confirmed that mice deficient in microglia exhibit reduced antiviral cytokine secretion and enhanced viral replication during early HSV-1 infection [55], demonstrating that microglia contribute significantly to controlling viral spread within the CNS. Microglia employ distinct TLR signaling pathways that serve either protective or pathogenic roles. TLR3 plays a critical role in type I IFNs induction; its deficiency impairs IFNs production and increases susceptibility to HSE [56]. Mechanistically, TLR3 detects viral dsRNA intermediates and recruits the adaptor protein TRIF, which in turn activates IRF3 to drive IFN‑β expression. In contrast to the protective role of TLR3, TLR2 activation in microglia drives pro‑inflammatory cytokine production. Studies have shown that HSV-1 infection upregulates TLR2 expression and activates the MyD88‑dependent pathway in BV2 microglial cells, leading to NF-κB nuclear translocation and increased production of pro-inflammatory cytokines such as IL-6 and TNF-α. Knockdown of TLR2 with siRNA significantly reduces these cytokine responses, indicating that TLR2 activation is causally involved in microglial inflammatory cytokine production [57]. TLR9 has also been implicated in microglial inflammatory responses, as HSV-1 infection upregulates TLR9 expression and promotes cytokine production in BV2 cells [58]; however, direct evidence that TLR9 activation drives these responses remains lacking.

Astrocytes also participate in HSV recognition and immune regulation via TLRs. TLR3 plays a critical role in astrocyte antiviral defense. Following HSV-2 infection, primary astrocytes derived from TLR3-deficient mice exhibit impaired type I IFNs production and elevated viral replication compared to wild-type astrocytes, indicating that TLR3 is required for the intrinsic antiviral response in this cell type [59]. In contrast to the protective role of TLR3, HSV‑1 infection induces the expression of TLR2 and TLR4 in astrocytes, which promotes neuroinflammation [60]. These effects depend on productive viral replication, as treatment with acyclovir abrogates TLR induction. Mechanistically, TLR2 and TLR4 activation triggers the production of pro-inflammatory mediators, including IL-6 and the endogenous TLR2/TLR4 agonist serum amyloid A3 (SAA3), suggesting that TLR signaling may be amplified through locally produced endogenous ligands such as SAA3, thereby potentiating the inflammatory response in the CNS.

Thus, microglia and astrocytes employ distinct TLR signaling pathways that serve either protective or pathogenic roles. TLR3 mediates type I IFNs production and antiviral defense in both cell types [56,59]. In microglia, HSV-1 infection triggers TLR2 and TLR9 activation, which is associated with the production of pro‑inflammatory cytokines [57,58]. Likewise, HSV-1 infection in astrocytes activates TLR2 and TLR4, correlating with inflammatory responses [60]. Collectively, these findings establish a consistent link between HSV-1-induced TLR activation and CNS inflammation. Nevertheless, definitive evidence is required to delineate their specific contributions to the pathogenesis of HSV-1 encephalitis.

### Central neurons: the final target and the protective role of TLR3

The strength and integrity of TLR signaling in central neurons are critically associated with the outcome of CNS infection, determining susceptibility to acute encephalitis versus potential control of the virus [61]. The TLR3-mediated intrinsic immune response serves as a pivotal defense mechanism in these cells [62].

In cortical neurons, the TLR3 signaling pathway maintains constitutive levels of IFN-β expression, conferring a baseline intrinsic antiviral defense. Deficiency in TLR3 significantly increases the susceptibility of these central neurons to HSV-1 infection [63] (Figure 3). Human genetic evidence underscores this principle: a heterozygous variant in the E3 ubiquitin ligase WWP2 enhances TRIF ubiquitination, specifically impairing TLR3 signaling in neurons and thereby markedly increasing an individual’s susceptibility to HSE [64]. These findings collectively provide a molecular explanation for how congenital defects in the neuronal TLR3 pathway compromise intrinsic antiviral immunity, leading to uncontrolled viral replication and the pathogenesis of HSE in children [65,66].

In summary, within central neurons, robust TLR3 responses are crucial for resistance to lethal encephalitis, providing intrinsic antiviral defense through constitutive IFN-β expression.

A systematic overview of these cell-type-specific TLR responses is provided in Table 1.

**Table 1.** Cell-type-specific tlr expression, signaling, and functional outcomes in HSV infection.

Cell type	TLRs involved	Key adaptors	Downstream outputs	Biological outcome	References
Epithelial Cells	TLR4	MyD88, TRIF	Early (4–6 h): activation of NF-κB and IRF3/7 leads to IFN-β and IL-6 production; Late (24 h): activation of AP-1 leads to TLR4 upregulation	Early antiviral defense; later exploited by virus (AP-1 positive feedback)	[26–28]
	TLR9	MyD88	Activation of NF-κB	Antiviral effects observed under certain conditions; Exploited by virus via NF-κB to enhance viral gene transcription	[29–33]
Fibroblasts	TLR2, TLR3, TLR7	/	Inflammatory cytokines	Early antiviral state	[35]
Plasmacytoid DCs	TLR7, TLR9	MyD88	IRF7; high levels of type I IFNs	Systemic antiviral state, viral clearance	[40,42]
Macrophages	TLR9, TLR2	MyD88, TRIF, Mal	TLR9: IFN-β, TNF-α; TLR2 and TLR9 collectively: iNOS-mediated NO production	Viral clearance	[44–48]
Trigeminal Ganglion Neurons	TLR3(attenuated)	/	Subdued IFNs response	Permissive for latency	[51]
Enteric Neurons	TLR2	MyD88	CCL2, pro-inflammatory mediators	Local pathology, neuromuscular dysfunction	[53,54]
Microglia	TLR3	TRIF	IFN-β	antiviral defense	[56]
	TLR2, TLR9	MyD88	IL-6, TNF-α, MCP-1	pathogenic inflammation	[57,58]
Astrocytes	TLR3	TRIF	Type I IFNs	antiviral defense	[59]
	TLR2, TLR4	MyD88	IL-6, SAA3	neuroinflammation	[60]
Cortical Neurons	TLR3	TRIF	Constitutive IFN-β	Intrinsic antiviral defense, protection against HSE	[62,63]

## Concluding remarks and future perspectives

### A spatiotemporal model of TLR-Mediated immunity

**Figure 4.**
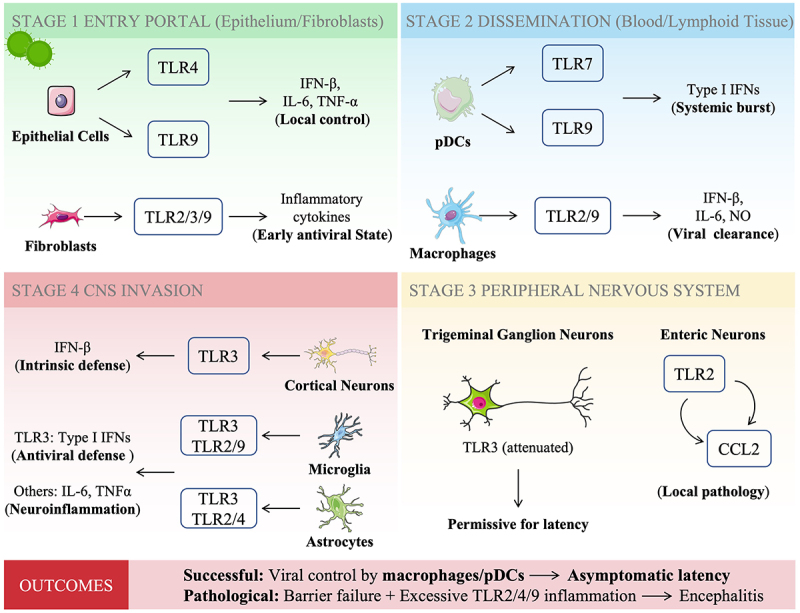
Spatiotemporal model of TLR-mediated immunity during HSV pathogenesis. The schematic illustrates the journey of HSV from the periphery to the CNS, highlighting the dominant tlr responses at each anatomical site.

Synthesizing the cell-type-specific findings discussed above, Figure 4 presents a spatiotemporal model of TLR-mediated immunity, tracing the virus from peripheral entry to CNS invasion. At the initial portal of entry, epithelial cells utilize TLR4 and TLR9 to initiate local antiviral responses [26,27], while fibroblasts contribute through TLR2, TLR3, and TLR7 [29]. During dissemination, pDC mount a systemic type I IFNs burst through TLR7/9 [40], while macrophages mediate viral recognition primarily via TLR9 with contributory roles for TLR2 [45,47,48]. As the infection reaches the nervous system, outcome is determined by cell-type-specific TLR responses. In peripheral sensory neurons, attenuated TLR3 signaling permits latency establishment [51]. In contrast, central neurons rely on robust TLR3-mediated intrinsic immunity as the final barrier against encephalitis. Meanwhile, microglia and astrocytes regulate neuroinflammation through distinct but partially overlapping TLR profiles [55,58–60], whereas TLR3 in both cell types contributes to antiviral defense [56,59]. The balance between effective viral control at each anatomical barrier and excessive inflammatory responses determines clinical outcome, ranging from asymptomatic latency to fatal encephalitis.

A complementary perspective to this spatiotemporal framework is provided by serotype-specific TLR engagement. HSV-1 and HSV-2 differ in their clinical manifestations, tissue tropism, and neuropathogenicity [67], raising the question of whether these differences are reflected in distinct TLR engagement across cell types. Table 2 consolidates the available evidence on TLR involvement in HSV-1 versus HSV-2 infection across key cell types, providing a framework for identifying potential serotype-specific patterns. However, most studies to date have focused on a single serotype, making direct comparisons challenging and highlighting a significant gap in the field.

**Table 2.** Comparative involvement of TLRs in HSV-1 vs. HSV-2 infection.

TLR	Cell type	Role in HSV-1	Role in HSV-2	Evidence strength
TLR2	Enteric neurons	Triggers TLR2-MyD88 signaling, CCL2 production, neuromuscular dysfunction [53,54]	Currently unclear	TLR2 knockout ameliorates gastrointestinal motility disorders
	Microglia	Detrimental (excessive inflammation) [57]	Currently unclear	Pathogenic in CNS
	Astrocytes	Induced upon HSV-1 infection, promotes neuroinflammation [60]	Currently unclear	Upregulated with TLR4; contributes to inflammatory response
TLR3	Trigeminal ganglion neurons	Attenuated TLR3-dependent immune responses [51]	Currently unclear	May facilitate latency establishment
	Microglia	Deficiency impairs IFN-β production [56]	Currently unclear	Protective role in antiviral defense
	Astrocytes	Currently unclear	Required for type I IFNs production [59]	TLR3^−^ /^−^ astrocytes exhibit impaired IFN-β and elevated viral replication
	Cortical neurons	Critical for CNS defense; maintains constitutive IFN-β expression; deficiency increases susceptibility [63]	Currently unclear	Well-established for HSV-1 CNS defense
TLR4	Epithelial cells	Currently unclear	Early (4–6 h): antiviral (IFN-β, IL-6) [26,27]; Late (24 h): exploited via AP-1 positive feedback [28]	More prominently studied in HSV-2 genital models
TLR9	Epithelial cells	Currently unclear	Upregulated via SP1/JNK [30]	Virus exploits NF-κB downstream [31]
	pDCs	Induces type I IFNs [42]	Currently unclear	/
	Macrophages	Induces IFN-β and TNF-α via Mal [45]; synergizes with TLR2 for viral control [47,48]	Currently unclear	*In vivo* evidence from TLR2/9 double-knockout mice

### Translational opportunities and challenges in TLR-based therapeutics

Beyond understanding basic mechanisms, there is significant translational potential in exploiting TLR modulation for therapeutic benefit [68]. Preclinical studies have provided proof-of-concept for this approach; for instance, intranasal administration of the TLR3 agonist poly(I:C) or TLR9 agonist oligodeoxynucleotides (ODN) significantly improved survival in a mouse model of HSV-1 encephalitis by reducing viral load and inducing early immune gene expression in the brain [69,70]. Similarly, the TLR7 agonist SMIP-7.7 has been shown to significantly reduce vaginal viral titers in a guinea pig model of genital HSV-2 disease [71]. This preclinical promise has spurred interest in several clinical candidates. The TLR7 agonist imiquimod and the TLR7/8 agonist resiquimod have been evaluated for the topical treatment of HSV infections [72]. However, despite case reports documenting successful treatment of HSV lesions with imiquimod [73,74], formal clinical trials have demonstrated inconsistent efficacy. A phase II study of imiquimod 5% cream in patients with recurrent genital herpes failed to show a significant reduction in recurrence rates compared with placebo [75]. Clinical evaluation of resiquimod has similarly yielded disappointing results, with a phase II trial finding no significant effect on lesion healing or shedding duration [76]. These findings underscore the considerable gap between laboratory discovery and clinical efficacy.

To bridge this gap, advanced drug delivery systems are being developed to enhance efficacy while minimizing off-target effects. For example, liposome-dual TLR agonist complex (LTAC) nanoparticles have been designed as a local ocular immunotherapy [77]. These nanoparticles consist of cationic liposomes complexed with a TLR3 agonist (polyinosinic, polycytidylic acid) and a TLR9 agonist (non-coding plasmid DNA). The cationic liposomes facilitate the entry of nucleic acid agonists into endosomal compartments where TLR3 and TLR9 are expressed [78], while the inclusion of carboxymethyl cellulose enhances adhesion to epithelial cells of the conjunctiva and cornea. In preclinical models, ocular administration of LTAC nanoparticles led to rapid symptom relief, including the resolution of keratitis and corneal ulcers.

Despite these advances, a major hurdle in translating TLR-based therapies is the “double-edged sword” nature of TLR modulation. While TLR9 activation can reduce viral load, its excessive stimulation has also been linked to exacerbated neuroinflammation in some models of HSV encephalitis [70]. Therefore, systemic administration of TLR agonists carries inherent risks of triggering autoimmunity or uncontrolled inflammation. This necessitates the development of even more refined strategies, such as cell-specific delivery systems (e.g. nanoparticle-based carriers targeting pDCs or CNS-resident cells) or the strategic use of TLR antagonists to dampen immunopathology during the post-infection phase.

### Future directions

Current studies have begun to elucidate how HSV induces the expression of various TLRs to activate downstream inflammatory and interferon pathways in different cells. However, existing knowledge is heavily biased toward a limited set of well-characterized TLR-cell combinations, such as TLR3 in cortical neurons and TLR9 in pDCs. In contrast, many potential interactions remain poorly understood. For instance, the roles of TLR2 in trigeminal ganglion neurons, TLR7 in macrophages, and TLR8 in any cell type during HSV infection have yet to be explored. This imbalance highlights the need for systematic future studies using cell-type-specific knockout models and comparative approaches across both HSV serotypes.

Given that HSV treatment is still constrained by drug resistance, further exploration of TLRs in HSV immune recognition and evasion holds substantial scientific and clinical importance. Future research should focus on investigating the mechanisms by which HSV-encoded proteins target and suppress TLR signaling pathways, particularly the interferon induction cascade, to reveal strategies of virus-host co-evolution. There is also significant potential in exploiting TLR agonists, such as those for TLR3, TLR7/8, and TLR9, as vaccine adjuvants to enhance the immunogenicity of HSV vaccines or for topical application to boost local immune clearance in mucosal infections [79,80]. Furthermore, studying the regulatory mechanisms of TLR signaling in neurons may reveal whether modulating these pathways can impact the latent viral reservoir, offering novel approaches to clear persistent infection. By systematically clarifying the network of TLR functions across different cell types during HSV infection, this review not only deepens the understanding of herpesvirus pathogenesis but also provides a critical theoretical foundation for developing new immunotherapies and vaccines.

## Data Availability

There is no data associated with this research.

## References

[1] Birkmann A, Saunders R. Overview on the management of herpes simplex virus infections: current therapies and future directions. Antiviral Res. 2025;237:106152. doi: 10.1016/j.antiviral.2025.106152 40154924

[2] Kalamvoki M. HSV-1 virions and related particles: biogenesis and implications in the infection. J Virol. 2025;99(3):e0107624. doi: 10.1128/jvi.01076-24 39898651 PMC11915793

[3] Jones C. Human alpha-herpesvirus 1 (HSV-1) viral replication and reactivation from latency are expedited by the glucocorticoid receptor. J Virol. 2025;99(4):e0030325. doi: 10.1128/jvi.00303-25 40145740 PMC11998515

[4] Kuang S, He Z, Zhang J, et al. HSV-1 hijacks mitochondrial dynamics: potential molecular mechanisms linking viral infection to neurodegenerative disorders. Apoptosis. 2025;30(9–10):1913–13. doi: 10.1007/s10495-025-02142-9 40593393

[5] Owen EM, Jama M, Nahal B, et al. 20 years of herpes simplex virus type 2 (HSV-2) research in low-income and middle-income countries: systematic evaluation of progress made in addressing who priorities for research in HSV-2/HIV interactions, HSV-2 control and mathematical modelling. BMJ Glob Health. 2024;9(7):e015167. doi: 10.1136/bmjgh-2024-015167 PMC11227757 38964882

[6] Pierce CA, Loh LN, Steach HR, et al. HSV-2 triggers upregulation of MALAT1 in CD4+ T cells and promotes HIV latency reversal. J Clin Invest. 2023;133(11):e164317. doi: 10.1172/JCI164317 37079384 PMC10232005

[7] Chen R, Zou J, Chen J, et al. Pattern recognition receptors: function, regulation and therapeutic potential. Signal Transduct Target Ther. 2025;10(1):216. doi: 10.1038/s41392-025-02264-1 40640149 PMC12246121

[8] Campos MA, Zolini GP, Kroon EG. Impact of toll-like receptors (TLRs) and tlr signaling proteins in trigeminal ganglia impairing herpes simplex virus 1 (HSV-1) progression to encephalitis: insights from mouse models. Front Biosci (Landmark Ed). 2024;29(3):102. 38538263 10.31083/j.fbl2903102

[9] Kayesh MEH, Kohara M, Tsukiyama-Kohara K. Tlr agonists as vaccine adjuvants in the prevention of viral infections: an overview. Front Microbiol. 2023;14:1249718. 38179453 10.3389/fmicb.2023.1249718 PMC10764465

[10] Kim BS. Critical role of tlr activation in viral replication, persistence, and pathogenicity of Theiler’s virus. Front Immunol. 2023;14:1167972. doi: 10.3389/fimmu.2023.1167972 37153539 PMC10157096

[11] Chen L, Zheng L, Chen P, et al. Myeloid differentiation primary response protein 88 (MyD88): the central hub of TLR/IL-1R signaling. J Med Chem. 2020;63(22):13316–13329. doi: 10.1021/acs.jmedchem.0c00884 32931267

[12] Manik MK, Pan M, Xiao L, et al. Structural basis for TIR domain-mediated innate immune signaling by Toll-like receptor adaptors TRIF and tram. Proc Natl Acad Sci USA. 2025;122(2):e2418988122. 39786929 10.1073/pnas.2418988122 PMC11745336

[13] Feng E, Balint E, Vahedi F, et al. Immunoregulatory functions of interferons during genital HSV-2 infection. Front Immunol. 2021;12:724618. doi: 10.3389/fimmu.2021.724618 34484233 PMC8416247

[14] Lind NA, Rael VE, Pestal K, et al. Regulation of the nucleic acid-sensing Toll-like receptors. Nat Rev Immunol. 2022;22(4):224–235. doi: 10.1038/s41577-021-00577-0 34272507 PMC8283745

[15] Zhong X, Zhang Y, Yuan M, et al. Prunella vulgaris polysaccharide inhibits herpes simplex virus infection by blocking TLR-mediated NF-κB activation. Chin Med. 2024;19(1):6. doi: 10.1186/s13020-023-00865-y 38185640 PMC10773030

[16] Latif MB, Raja R, Kessler PM, et al. Relative contributions of the cGAS-STING and TLR3 signaling pathways to attenuation of herpes simplex virus 1 replication. J Virol. 2020;94(6):e01717–19. doi: 10.1128/JVI.01717-19 31896590 PMC7158705

[17] Tucker MH, Kalamvoki M, Tilak K, et al. The immunogenetic basis of severe herpes simplex infections in neonates and children: a review. Pediatr Res. 2025;97(4):1370–1380. doi: 10.1038/s41390-025-03830-7 39827257

[18] Tal Y, Ribak Y, Khalaila A, et al. Toll-like receptor 3 (TLR3) variant and NLRP12 mutation confer susceptibility to a complex clinical presentation. Clin Immunol. 2020;212:108249. doi: 10.1016/j.clim.2019.108249 31445170

[19] Li LJ, Zhang SJ, Liu P, et al. Corilagin interferes with Toll-like receptor 3-mediated immune response in herpes simplex encephalitis. Front Mol Neurosci. 2019;12:83. doi: 10.3389/fnmol.2019.00083 31080403 PMC6497770

[20] Wong SW, Pockar S, Steeples LR, et al. Bilateral sequential herpes simplex type 2 panophthalmitis in an adult with a Toll-like receptor 4 mutation. Ocul Immunol Inflamm. 2025;33(8):1886–1889. doi: 10.1080/09273948.2025.2524586 40657869

[21] Zhu H, Zheng C. The race between host antiviral innate immunity and the immune evasion strategies of herpes simplex virus 1. Microbiol Mol Biol Rev. 2020;84(4):e0010323. doi: 10.1128/MMBR.00099-20 PMC7528619 32998978

[22] Lenart M, Działo E, Kluczewska A, et al. miRNA regulation of NK cells antiviral response in children with severe and/or recurrent herpes simplex virus infections. Front Immunol. 2020;11:589866. 33679688 10.3389/fimmu.2020.589866 PMC7931645

[23] Chan T, Barra NG, Lee AJ, et al. Innate and adaptive immunity against herpes simplex virus type 2 in the genital mucosa. J Reprod Immunol. 2011;88(2):210–218. doi: 10.1016/j.jri.2011.01.001 21334750

[24] Tognarelli EI, Palomino TF, Corrales N, et al. Herpes simplex virus evasion of early host antiviral responses. Front Cell Infect Microbiol. 2019;9:127. doi: 10.3389/fcimb.2019.00127 31114761 PMC6503643

[25] Sharma P, Naqvi RA, Borase H, et al. Global MicroRNA profiling of HSV-1 infected cornea identifies miR-329 as a novel regulator of virus infection. Invest Ophthalmol Vis Sci. 2025;66(2):61. doi: 10.1167/iovs.66.2.61 PMC11878248 39992671

[26] Liu H, Chen K, Feng W, et al. TLR4-MyD88/Mal-NF-kB axis is involved in infection of HSV-2 in human cervical epithelial cells. PLOS ONE. 2013;8(11):e80327. doi: 10.1371/journal.pone.0080327 24278275 PMC3835891

[27] Liu H, Chen K, Feng W, et al. HSV-2 increases TLR4-dependent phosphorylated IRFs and IFN-β induction in cervical epithelial cells. PLOS ONE. 2014;9(4):e94806. doi: 10.1371/journal.pone.0094806 24722640 PMC3983257

[28] Lv X, Wang H, Su A, et al. Herpes simplex virus type 2 infection triggers AP-1 transcription activity through TLR4 signaling in genital epithelial cells. Virol J. 2018;15(1):173. doi: 10.1186/s12985-018-1087-3 30419930 PMC6233380

[29] Shi J, Kuang L, Qi L, et al. Effect of the TLR9 signaling pathway on acyclovir infection with herpes simplex virus type 2 in HaCaT cells. Front Microbiol. 2025;16:1560340. doi: 10.3389/fmicb.2025.1560340 40196029 PMC11974507

[30] Hu K, Fu M, Wang J, et al. HSV-2 infection of human genital epithelial cells upregulates TLR9 expression through the SP1/JNK signaling pathway. Front Immunol. 2020;11:356. doi: 10.3389/fimmu.2020.00356 32194565 PMC7065266

[31] Zhou HY, Gao SQ, Gong YS, et al. Anti-HSV-1 effect of dihydromyricetin from Ampelopsis grossedentata via the TLR9-dependent anti-inflammatory pathway. J Glob Antimicrob Resist. 2020;23:370–376. doi: 10.1016/j.jgar.2020.10.003 33161114

[32] Lee S, Lee HH, Shin YS, et al. The anti-HSV-1 effect of quercetin is dependent on the suppression of TLR-3 in raw 264.7 cells. Arch Pharm Res. 2017;40(5):623–630. doi: 10.1007/s12272-017-0898-x 28258480

[33] Hung PY, Ho BC, Lee SY, et al. Houttuynia cordata targets the beginning stage of herpes simplex virus infection. PLOS ONE. 2015;10(2):e0115475. doi: 10.1371/journal.pone.0115475 25643242 PMC4314066

[34] Chansard A, Dubrulle N, Poujol de Molliens M, et al. Corrigendum: unveiling interindividual variability of human fibroblast innate immune response using robust cell-based protocols. Front Immunol. 2021;12:685768. doi: 10.3389/fimmu.2021.685768 33981321 PMC8109174

[35] Zhang Y, Lo K, Wang C, et al. Herpes simplex virus-induced upregulation of inflammatory cytokines in human gingival fibroblasts. Virol J. 2024;21(1):323. doi: 10.1186/s12985-024-02595-5 39702408 PMC11660554

[36] Yao XD, Rosenthal KL. Herpes simplex virus type 2 virion host shutoff protein suppresses innate dsRNA antiviral pathways in human vaginal epithelial cells. J Gen Virol. 2011;92(Pt 9):1981–1993. 21632561 10.1099/vir.0.030296-0

[37] Krug A, Luker GD, Barchet W, et al. Herpes simplex virus type 1 activates murine natural interferon-producing cells through toll-like receptor 9. Blood. 2004;103(4):1433–1437. doi: 10.1182/blood-2003-08-2674 14563635

[38] Lund J, Sato A, Akira S, et al. Toll-like receptor 9-mediated recognition of herpes simplex virus-2 by plasmacytoid dendritic cells. J Exp Med. 2003;198(3):513–520. 12900525 10.1084/jem.20030162 PMC2194085

[39] Barrow AD, Cella M, Edeling MA, et al. Cutting edge: PDGF-DD binding to NKp44 costimulates TLR9 signaling and proinflammatory cytokine secretion in human plasmacytoid dendritic cells. J Immunol. 2024;212(3):369–374. 38117750 10.4049/jimmunol.2200496

[40] Kawai T, Sato S, Ishii KJ, et al. Interferon-alpha induction through Toll-like receptors involves a direct interaction of IRF7 with MyD88 and TRAF6. Nat Immunol. 2004;5(10):1061–1068. doi: 10.1038/ni1118 15361868

[41] Cai M, Huang W, Hu X, et al. MEKK3 activates IRF7 to trigger a potent type I interferon induction in response to TLR7/9 signaling. Mol Immunol. 2021;134:183–191. doi: 10.1016/j.molimm.2021.03.008 33812250

[42] Jamali A, Hu K, Sendra VG, et al. Characterization of resident corneal plasmacytoid dendritic cells and their pivotal role in herpes simplex keratitis. Cell Rep. 2020;32(9):108099. doi: 10.1016/j.celrep.2020.108099 32877681 PMC7511260

[43] Ding X, Lai X, Klaestrup IH, et al. Temporally resolved single-cell RNA sequencing reveals protective and pathological responses during herpes simplex virus CNS infection. J Neuroinflammation. 2025;22(1):146. doi: 10.1186/s12974-025-03471-x 40450318 PMC12125739

[44] Lang J, Bohn P, Bhat H, et al. Acid ceramidase of macrophages traps herpes simplex virus in multivesicular bodies and protects from severe disease. Nat Commun. 2020;11(1):1338. doi: 10.1038/s41467-020-15072-8 32165633 PMC7067866

[45] Zyzak J, Mitkiewicz M, Leszczyńska E, et al. HSV-1/TLR9-mediated IFNβ and TNFα induction is Mal-dependent in macrophages. J Innate Immun. 2020;12(5):387–398. doi: 10.1159/000504542 31851971 PMC7506264

[46] Hartenian E, Agustoni M, Broz P. NINJ1 blocks HSV-1 entry into macrophages to impact viral replication and immunity. EMBO Rep. 2026;27(1):69–88. doi: 10.1038/s44319-025-00638-8 41261285 PMC12796307

[47] Zolini GP, Lima GK, Lucinda N, et al. Defense against HSV-1 in a murine model is mediated by iNOS and orchestrated by the activation of TLR2 and TLR9 in trigeminal ganglia. J Neuroinflammation. 2014;11(1):20. doi: 10.1186/1742-2094-11-20 24479442 PMC3922087

[48] Lima GK, Zolini GP, Mansur DS, et al. Toll-like receptor (tlr) 2 and TLR9 expressed in trigeminal ganglia are critical to viral control during herpes simplex virus 1 infection. Am J Pathol. 2010;177(5):2433–2445. doi: 10.2353/ajpath.2010.100121 20864677 PMC2966801

[49] van Lint AL, Murawski MR, Goodbody RE, et al. Herpes simplex virus immediate-early ICP0 protein inhibits Toll-like receptor 2-dependent inflammatory responses and NF-kappaB signaling. J Virol. 2010;84(20):10802–10811. doi: 10.1128/JVI.00063-10 20686034 PMC2950559

[50] Peri P, Mattila RK, Kantola H, et al. Herpes simplex virus type 1 Us3 gene deletion influences toll-like receptor responses in cultured monocytic cells. Virol J. 2008;5(1):140. doi: 10.1186/1743-422X-5-140 19025601 PMC2605447

[51] Zimmer B, Ewaleifoh O, Harschnitz O, et al. Human iPSC-derived trigeminal neurons lack constitutive TLR3-dependent immunity that protects cortical neurons from HSV-1 infection. Proc Natl Acad Sci USA. 2018;115(37):E8775–e8782. doi: 10.1073/pnas.1809853115 30154162 PMC6140487

[52] Zhu S, Viejo-Borbolla A. Pathogenesis and virulence of herpes simplex virus. Virulence. 2021;12(1):2670–2702. doi: 10.1080/21505594.2021.1982373 34676800 PMC8923070

[53] Bansode YD, Chattopadhyay D, Saha B. Transcriptomic analysis of interferon response in Toll-like receptor 2 ligand-treated and herpes simplex virus 1-infected neurons and astrocytes. Viral Immunol. 2021;34(4):256–266. doi: 10.1089/vim.2020.0238 33351727

[54] Brun P, Scarpa M, Marchiori C, et al. Herpes simplex virus type 1 engages Toll like receptor 2 to recruit macrophages during infection of enteric neurons. Front Microbiol. 2018;9:2148. doi: 10.3389/fmicb.2018.02148 30254622 PMC6141724

[55] Katzilieris-Petras G, Lai X, Rashidi AS, et al. Microglia activate early antiviral responses upon herpes simplex virus 1 entry into the brain to counteract development of encephalitis-like disease in mice. J Virol. 2022;96(6):e0131121. doi: 10.1128/jvi.01311-21 35045263 PMC8941881

[56] Liu J, Chen X, Liu J, et al. HSV-1 immune escapes in microglia by down-regulating GM130 to inhibit TLR3-mediated innate immune responses. Virol J. 2024;21(1):219. doi: 10.1186/s12985-024-02492-x 39285274 PMC11404012

[57] Guo YJ, Luo T, Wu F, et al. Corilagin protects against HSV1 encephalitis through inhibiting the TLR2. Mol Neurobiol. 2015;52(3):1547–1560. doi: 10.1007/s12035-014-8947-7 25367881

[58] Guo YJ, Luo T, Wu F, et al. Involvement of TLR2 and TLR9 in the anti-inflammatory effects of chlorogenic acid in HSV-1-infected microglia. Life Sci. 2015;127:12–18. doi: 10.1016/j.lfs.2015.01.036 25744394

[59] Reinert LS, Harder L, Holm CK, et al. TLR3 deficiency renders astrocytes permissive to herpes simplex virus infection and facilitates establishment of CNS infection in mice. J Clin Invest. 2012;122(4):1368–1376. doi: 10.1172/JCI60893 22426207 PMC3314467

[60] Villalba M, Hott M, Martin C, et al. Herpes simplex virus type 1 induces simultaneous activation of Toll-like receptors 2 and 4 and expression of the endogenous ligand serum amyloid a in astrocytes. Med Microbiol Immunol. 2012;201(3):371–379. doi: 10.1007/s00430-012-0247-0 22622619

[61] Harschnitz O, Studer L. Human stem cell models to study host–virus interactions in the central nervous system. Nat Rev Immunol. 2021;21(7):441–453. doi: 10.1038/s41577-020-00474-y 33398129 PMC9653304

[62] Manglani M, Poley M, Kumar A, et al. Anti-NMDAR encephalitis after neonatal HSV-1 infection in a child with low TLR-3 function. Pediatrics. 2021;148(3):e2020035824. doi: 10.1542/peds.2020-035824 34385350

[63] Gao D, Ciancanelli MJ, Zhang P, et al. TLR3 controls constitutive IFN-β antiviral immunity in human fibroblasts and cortical neurons. J Clin Invest. 2021;131(1):e134529. doi: 10.1172/JCI134529 33393505 PMC7773389

[64] Bibert S, Quinodoz M, Perriot S, et al. Herpes simplex encephalitis due to a mutation in an E3 ubiquitin ligase. Nat Commun. 2024;15(1):3969. doi: 10.1038/s41467-024-48287-0 38730242 PMC11087577

[65] Lafaille FG, Pessach IM, Zhang SY, et al. Impaired intrinsic immunity to HSV-1 in human iPSC-derived TLR3-deficient CNS cells. Nature. 2012;491(7426):769–773. doi: 10.1038/nature11583 23103873 PMC3527075

[66] Zhang SY, Casanova JL. Genetic defects of brain immunity in childhood herpes simplex encephalitis. Nature. 2024;635(8039):563–573. doi: 10.1038/s41586-024-08119-z 39567785 PMC11822754

[67] Andreu S, Galdo-Torres D, Ripa I, et al. From HSV-2 to HSV-1: a change in the epidemiology of genital herpes. J Infect. 2025;91(5):106636. doi: 10.1016/j.jinf.2025.106636 41115532

[68] Bhagchandani S, Johnson JA, Irvine DJ. Evolution of Toll-like receptor 7/8 agonist therapeutics and their delivery approaches: from antiviral formulations to vaccine adjuvants. Adv Drug Deliv Rev. 2021;175:113803. doi: 10.1016/j.addr.2021.05.013 34058283 PMC9003539

[69] Boivin N, Sergerie Y, Rivest S, et al. Effect of pretreatment with toll-like receptor agonists in a mouse model of herpes simplex virus type 1 encephalitis. J Infect Dis. 2008;198(5):664–672. doi: 10.1086/590671 18662130

[70] Boivin N, Menasria R, Piret J, et al. Modulation of TLR9 response in a mouse model of herpes simplex virus encephalitis. Antiviral Res. 2012;96(3):414–421. doi: 10.1016/j.antiviral.2012.09.022 23043942

[71] Bernstein DI, Cardin RD, Bravo FJ, et al. Topical SMIP-7.7, a toll-like receptor 7 agonist, protects against genital herpes simplex virus type-2 disease in the Guinea pig model of genital herpes. Antivir Chem Chemother. 2014;23(5):189–196. 23232327 10.3851/IMP2499

[72] Miller RL, Meng TC, Tomai MA. The antiviral activity of Toll-like receptor 7 and 7/8 agonists. Drug News Perspect. 2008;21(2):69–87. 18389099 10.1358/dnp.2008.21.2.1188193

[73] Deza G, Martin-Ezquerra G, Curto-Barredo L, et al. Successful treatment of hypertrophic herpes simplex genitalis in HIV-infected patient with topical imiquimod. J Dermatol. 2015;42(12):1176–1178. doi: 10.1111/1346-8138.12969 26074211

[74] Sachan S, Potluri VK, Gopinath H, et al. Recalcitrant ulcerative genital herpes in an immunocompetent individual treated successfully with imiquimod. Int J STD AIDS. 2024;35(3):231–233. doi: 10.1177/09564624231213110 37938029

[75] Schacker TW, Conant M, Thoming C, et al. Imiquimod 5-percent cream does not alter the natural history of recurrent herpes genitalis: a phase ii, randomized, double-blind, placebo-controlled study. Antimicrob Agents Chemother. 2002;46(10):3243–3248. doi: 10.1128/AAC.46.10.3243-3248.2002 12234851 PMC128805

[76] Fife KH, Meng TC, Ferris DG, et al. Effect of resiquimod 0.01% gel on lesion healing and viral shedding when applied to genital herpes lesions. Antimicrob Agents Chemother. 2008;52(2):477–482. doi: 10.1128/AAC.01173-07 18039918 PMC2224757

[77] Lappin M, Wotman K, Chow L, et al. Nanoparticle ocular immunotherapy for herpesvirus surface eye infections evaluated in cat infection model. PLOS ONE. 2023;18(1):e0279462. doi: 10.1371/journal.pone.0279462 36607992 PMC9821494

[78] Erikçi E, Gursel M, Gürsel I. Differential immune activation following encapsulation of immunostimulatory CpG oligodeoxynucleotide in nanoliposomes. Biomaterials. 2011;32(6):1715–1723. doi: 10.1016/j.biomaterials.2010.10.054 21112627

[79] Cao H, Cao Z, Han Y, et al. Next-generation sequencing and immuno-informatics for designing a multi-epitope vaccine against HSV-1-induced uveitis. Front Immunol. 2025;16:1461725. doi: 10.3389/fimmu.2025.1461725 39958333 PMC11825787

[80] Hasan M, Islam S, Chakraborty S, et al. Contriving a chimeric polyvalent vaccine to prevent infections caused by herpes simplex virus (type-1 and type-2): an exploratory immunoinformatic approach. J Biomol Struct Dyn. 2020;38(10):2898–2915. doi: 10.1080/07391102.2019.1647286 31328668

